# Comparison of Intestinal Microbiota Between Healthy and MMVD Chihuahuas Using 16S rRNA Gene Amplicon Sequencing

**DOI:** 10.3389/fvets.2022.846492

**Published:** 2022-03-30

**Authors:** Ryuji Araki, Koji Iwanaga, Kazunori Ueda, Ayaka Shima, Genki Ishihara, Mitsuhiro Aizu, Toshiharu Fukayama, Mitsuhiro Isaka

**Affiliations:** ^1^Yokohama Yamate Dog & Cat Medical Center, Yokohama, Japan; ^2^Tokyo Veterinary Cardiology Center, Tokyo, Japan; ^3^Department of Small Animal Clinical Sciences, School of Veterinary Medicine, Rakuno Gakuen University, Ebetsu, Japan; ^4^Anicom Insurance, Inc., Tokyo, Japan

**Keywords:** Chihuahua, fractional shortening, intestinal complication, intestinal microbiota, myxomatous mitral valve disease

## Abstract

Myxomatous mitral valve disease (MMVD) is the most common cause of congestive heart failure in dogs, and although complications of MMVD to the lungs and kidneys have been identified, complications to the gut are less well understood. The intestinal microbiota is an important factor in the gut, and although the association between heart disease and the intestinal microbiota has been shown in human medicine, it is unknown in dogs. The study aimed to evaluate the relationship between MMVD and gut microbiota. A total of 69 healthy Chihuahuas and Chihuahuas with MMVD were evaluated for cardiac health by echocardiography and chest radiography and grouped according to ACVIM guidelines. Fecal samples were collected from all cases and 16S rRNA sequencing was used to reveal the intestinal microbiota. There were significant differences in LA/Ao, LVIDd, E vel, VHS, and VLAS with the severity of ACVIM. On the other hand, there were no significant differences in the diversity and composition of gut microbiota among the groups. The present study did not identify the effects of MMVD on the gut microbiota.

## Introduction

Much research has been done on the relationship between heart disease and the gut, and the concept of “Heart-Gut Axis” is well known. An important factor in understanding the function of the gut is the intestinal microbiota. In human medicine, it has been proven that the intestinal microbiota is involved in the pathogenesis of the cardiovascular disease, and it has been reported that lowering the blood concentration of trimethylamine N-oxide, a metabolite of intestinal bacteria, reduces the risk of cardiovascular disease (1–5). In addition, functional and morphological changes in the gut are induced in patients with heart failure, which in turn affects the gut microbiota. In both conditions, heart failure with preserved ejection fraction and heart failure with reduced ejection fraction, there were changes in the gut microbiota (6, 7). It has been reported that changes in the gut microbiota and the effects of heart failure on the intestinal tract can lead to systemic inflammation (8–12). Thus, in human medicine, the relationship between heart disease and intestinal microbiota has been the focus of much research and attention. Although research on the intestinal microbiota in veterinary medicine lags that in human medicine, it has been active in recent years due to the widespread use of 16S rRNA sequencing (13). The relationship between inflammatory bowel disease and intestinal microbiota has been reported, and the relationship between disease and intestinal microbiota has attracted attention in veterinary medicine, but there have been no reports on heart disease and intestinal microbiota (14).

MMVD is the most common cause of congestive heart failure in dogs. Cavalier King Charles Spaniels, Maltese, and Toy Poodles are reported to be the predominant breeds, and in clinical practice, MMVD is commonly seen in Chihuahuas due to many dogs kept. If MMVD worsens, it can cause fatal complications such as pulmonary edema and arrhythmia. It has been suggested that heart failure due to long-term MMVD can cause decreased blood flow throughout the body, leading to ischemic injury to vital organs such as the kidney and pancreas (15–17). We clarified the intestinal complications of MMVD using intestinal mucosal injury markers (I-FABP and D-Lactate) (18). To further understand the intestinal complications of MMVD, we investigated the fecal samples from MMVD-affected and healthy Chihuahuas using 16S rRNA gene sequencing to reveal the intestinal microbiota.

## Materials and Methods

### Animals

This study was approved by the Rakuno Gakuen University, School of Veterinary Medicine Institutional Animal Care and Use Committee (approval No. VH19A10). Dogs sampled at two institutions, Yokohama Yamate Dogs and Cats Medical Center and Tokyo Veterinary Cardiology Center, from April 2019 to May 2020 were included in the study. The breed was limited to Chihuahuas only. All Chihuahuas that met the following inclusion criteria and came to the hospital during the study period were included: (1) adult dogs; (2) consented to the study; (3) provided a fecal sample; (4) no cardiac disease not associated with MMVD; (5) no fatal comorbidities. Most of the healthy Chihuahuas came to the hospital for checkups and preventive medical care such as rabies vaccines. Patients with diseases secondary to MMVD (e.g., pulmonary hypertension or chronic kidney disease) were included; however, patients with other cardiac diseases (e.g., cardiac tumor or epicardial disease) were excluded. After obtaining the owner's consent, physical examination (weight and body condition score [BCS]), auscultation (presence and intensity of heart murmur), medical history, current medical history, medications, antibiotics, and the usual diet were recorded in all cases. In all the cases, chest radiography and echocardiography were performed to evaluate the heart.

### Heart Examination

In all cases, the heart was evaluated by chest X-ray and echocardiography. Echocardiography was performed according to standard techniques, using a Xario (TOSHIBA, probe: PST-50AT, 5MHz) at the Yokohama Yamate Dogs and Cats Medical Center and a LOGIC e9 (GE Health care Japan, probe: 6S) at the Tokyo Veterinary Cardiology Center (19). Data were collected by two examiners, and all examinations were performed in a quiet room. Echocardiography included the subjective evaluation of the valve structure and function; mitral regurgitation (MR) jets on color Doppler examination in four-lumen cross-sectional images, left atrium to aorta ratio (LA/Ao) at diastole, left ventricular septal wall thickness at diastole (IVSd), left ventricular posterior wall thickness at diastole (LVPWd), left ventricular internal diameter at diastole (LVIDd), normalized left ventricular internal diameter (LVIDDN) (20), left ventricular internal diameters in systole (LVIDs), fractional shortening (FS), left ventricular ejection fraction (LVEF), and left ventricular inflow velocity waves (Evel, Avel) were evaluated and recorded.Chest X-rays were taken at maximal right lateral inspiration for the presence of pulmonary edema and a recording of VHS, and VLAS was measured according to previous reports (21, 22). The ACVIM guidelines were used for the severity of MMVD (23).

### DNA Extraction From Fecal Samples

Fecal samples were collected using a specimen collection kit (Fecal collection container F, Fujifilm Wako Pharmaceutical) and sent to Anicom Insurance Inc. Add 200 μL of fecal samples and 810 μL of the lysis buffer (containing 224 μg/mL ProtenaseK) provided with the schematic kit to a Precellys 2 mL Soft Tissue Homogenizing Ceramic Beads Kit (KT03961-1-003.2, Bertin Instruments, France), and bead fragmentation (6,000 rpm, fragmentation 20 s, interval 30 s, fragmentation 20 s) was performed with a bead homogenizer Precellys Evolution (Bertin Instruments). The specimens were then lysed with Proteinase K by placing them on a heat block at 70°C for 10 min. Subsequently, Proteinase K was inactivated by placing the specimens on a heat block at 95°C for 5 min. The lysed samples were subjected to automated DNA extraction using chemagic 360 (PerkinElmer) and chemagic kit stool protocol to obtain 100 μL of DNA extract.

### 16S rRNA Gene Amplicon Sequencing

The DNAs were subjected to 16S rRNA gene amplicon sequencing. The V3–V4 regions of the 16S rRNA gene were amplified by PCR using the primers described in the Illumina 16S Sample Preparation Guide (Illumina, San Diego, CA, USA) as follows: forward, 5′-TCGTCGGCAGCGTCAGATGTGTATAAGAGACAG–CCTACGGGNGGCWGCAG-3′; reverse, 5′-GTCTCGTGGGCTCGGAGATGTGTATAAGAGACA-GGACTAC- HVGGGTATCTAATCC-3′. PCR amplification was performed using the KAPA HiFi HotStart Library Amplification Kit (Kapa Biosystems, Wilmington, MA, USA). Each PCR product was purified using an Agencourt AMPure XP Beads Kit (Beckman Coulter, Pasadena, CA, USA) and quantified using a Qubit dsDNA BR Assay Kit (Thermo Fisher Scientific, Waltham, MA, USA). One hundred nanograms of each amplicon were subjected to a second PCR round for indexing, using a Nextera XT Index Kit v2 (Illumina). After purification, the PCR products were quantified with a NanoPhotometer (Implen, Westlake Village, CA, USA) and pooled into one tube at a final concentration of 1.6 ng/μl. The concentration of the pooled DNA library was validated using an Agilent 2100 Bioanalyzer (Agilent, Santa Clara, CA, USA). After denaturation with NaOH,850 μl of 9pM DNA library and 150 μl of 9p M PhiX were mixed and subjected to pair-end sequencing using Illumina MiSeq with a MiSeq Reagent Kit v3 (600 cycles; Illumina).

### Processing of 16S rRNA Gene Amplicon Sequencing

The sequence data were processed using Quantitative Insights into Microbial Ecology 2 (QIIME 2) v2019.4.0 (24). The DADA2 software package v2019.4.0 incorporated in QIIME 2 was used to correct the amplicon sequence errors and to construct an amplicon sequence variant (ASV) table. The ASV table was rarefied. Microbial taxonomy was assigned using a Naïve Bayes classifier trained on the SILVA 132 99 % database.

### Microbial Diversity

Metrics of alpha diversity, including Shannon's index (Shannon), and those of beta diversity, including unweighted UniFrac and weighted UniFrac (weighted UniFrac), were examined using QIIME2. These diversity metrics were statistically analyzed by the method described below.

### Statistical Analysis

Case data were entered into a spreadsheet, and statistical analysis was performed using SPSS software (SPSS statistics ver 24.0 IBM Japan, Ltd., Tokyo, Japan). Age, weight, and BCS were subjected to a one-way analysis of variance using the Turley-Kramer *post-hoc* test. For gender and medications, Fisher's exact test was used. Values for each group obtained by echocardiography and chest radiography were subjected to a one-way analysis of variance using the Turley-Kramer *post-hoc* test.

## Results

### Animals

We collected a sample of 69 cases, ranging in age from 3 to 12 years, which were divided into four groups: Healthy chihuahuas (H) (*n* = 19), B1 (*n* = 21), B2 (*n* = 15), and C/D (*n* = 14) (Table 1). Of the 14 cases in Group C/D, 3 samples were taken at presentation to the hospital in a hypoxemic crisis of fulminant pulmonary edema. The remaining 11 cases were sampled on stable on medication at a scheduled recheck. There were no cases there that were excluded after sample collection. Group H had a predominantly lower mean age compared to the other groups. One case each in groups B1 and C/D was on antimicrobial medication. They were administered erythromycin and enrofloxacin, respectively. The following comorbidities were observed in each group. Group H: canine atopic dermatitis (CAD) (*n* = 2), chronic kidney disease (CKD) (*n* = 1), epileptic seizures (*n* = 1), and food allergy (*n* = 1). Group B1: CAD (*n* = 1), cholelithiasis (*n* = 1) hypoadrenocorticism (*n* = 1), and hypothyroidism (*n* = 2). Group B2: CAD (*n* = 1), CKD (*n* = 2), diabetes mellitus (*n* = 1), tracheal collapse (*n* = 2), and hypothyroidism (*n* = 1). Group C/D: CKD (*n* = 7), tracheal collapse (*n* = 2). Owing to worsening cardiac disease, many patients were taking cardiac medications, and all the patients in Group C/D were taking angiotensin-converting enzyme inhibitors (ACI) and pimobendan. The three patients in Group B2 on loop diuretics (furosemide: 1.1 ± 0.6 mg/kg/day) had no history of heart failure and, were being prescribed for cough reduction. Nine patients in Group C/D were taking loop diuretics (trasemide: 0.33 ± 0.23 mg/kg/day). The dietary content is summarized in Table 2.

**Table 1:** Patient characteristics of healthy Chihuahuas (H) and Chihuahuas with myxomatous mitral valve disease divided into three groups (B1, B2, and C/D).

	**H (*n* = 19)**	**B1 (*n* = 21)**	**B2 (*n* = 15)**	**C/D (*n* = 14)**
Age (years)	7.3 ± 2.6	11.1 ± 2.5	12.0 ± 2.4	11.8 ± 1.9
Sex (M/F)	9/10	11/10	6/9	8/6
Male/Casted male	0/9	2/9	2/4	3/5
Female/Spayed female	0/10	0/10	1/8	1/5
BW (kg)	3.1 ± 1.2	2.8 ± 1.0	3.0 ± 0.9	3.1 ± 1.0
Antibiotics	Nothing (*n* = 19)	Nothing (*n* = 20) Erythromycin (*n* = 1)	Nothing (*n* = 15)	Nothing (*n* = 13) Enrofloxacin (*n* = 1)
Comorbidities	CAD (*n* = 1) CKD (*n* = 1) Epileptic seizures (*n* = 1) Food allergy (n=1)	CAD (*n* = 1) Cholelithiasis (*n* = 1) Hypoadrenocorticism (*n* = 1) Hypothyroidism (*n* = 2)	CAD (*n* = 1) CKD (*n* = 2) Diabetes mellitus (*n* = 1) Hypothyroidism (*n* = 1) Tracheal collapse (*n* = 2)	CKD (*n* = 7) Tracheal collapse (*n* = 2)
**Medicine**				
ACE-I	*n* = 1	*n* = 2	*n* = 10	*n* = 14
Pimobendan	nothing	*n* = 1	*n* = 13	*n* = 14
Loop-diuretics	nothing	nothing	*n* = 3 (furosemide: 1.1 ± 0.6 mg/kg/day)	*n* = 9 (trasemide: 0.33 ± 0.23 mg/kg/day)

*Data are presented as mean ± standard deviation (SD).*

*BW, body weight; BCS, body condition score; CAD, canine atopic dermatitis; CKD, chronic kidney disease; ACE-I angiotensin-converting enzyme inhibitor*.

**Table 2:** Patient diet data in healthy Chihuahuas (H) and Chihuahuas with myxomatous mitral valve disease divided into three groups (B1, B2, and C/D).

**Group H (*n* = 19)**	**Group B1 (*n* = 21)**
Dry food, Royal canin, RENAL (*n* = 1)	Dry food, Royal canin, satiety renovation (*n* = 4)
Dry food, Royal canin, satiety renovation (*n* = 3)	Dry food, other companies, animal based (*n* = 2)
Dry food, Royal canin, Allergy (*n* = 3)	Dry food, Royal canin, Chihuahua 8 (*n* = 1)
Dry food, Royal canin, Urinary (*n* = 1)	Dry food, Royal canin, Gastrointestinal low fat (*n* = 5)
Dry food, Hill's, z/d (*n* = 2)	Dry food, other companies, animal based (n=6)
Dry food, other companies, animal based (*n* = 5)	Dry food, other companies, plant based (*n* = 1)
Dry food, other companies, plant based (n = 2)	Homemade food (*n* = 2)
Dry food, Royal canin, Skin support (*n* = 1)	Homemade food (*n* = 1)
**Group B2 (*****n*** **=** **15)**	**Group C/D (*****n*** **=** **14)**
Dry food, Royal canin, Chihuahua 8 (*n* = 3)	Dry food, Royal canin, RENAL (*n* = 4)
Dry food, Royal canin, Gastrointestinal low fat (*n* = 2)	Dry food, Royal canin, Gastrointestinal low fat (*n* = 2)
Dry food, Royal canin, Cardiac (*n* = 1)	Dry food, Royal canin, Neutered care (*n* = 1)
Dry food, Royal canin, satiety renovation (*n* = 1)	Dry food, Hill's, z/d (*n* = 1)
Dry food, Royal canin, RENAL (*n* = 2)	Dry food, other companies, animal based (*n* = 4)
Dry food, other companies, animal based (*n* = 6)	Homemade food (*n* = 2)

### Echocardiography and Chest Radiography

Echocardiography, VHS, and VLAS findings are presented in Table 2. LVIDd was higher in groups B2 and C/D than in groups H and B1; however, there was no significant difference between groups B2 and C/D. The E wave also increased with severity, with group C/D having the highest increase. VHS and VLAS were also higher with worsening cardiac disease, with Group C/D having higher values than the other groups.

### Intestinal Microbiota Analysis

**Figure 1:**
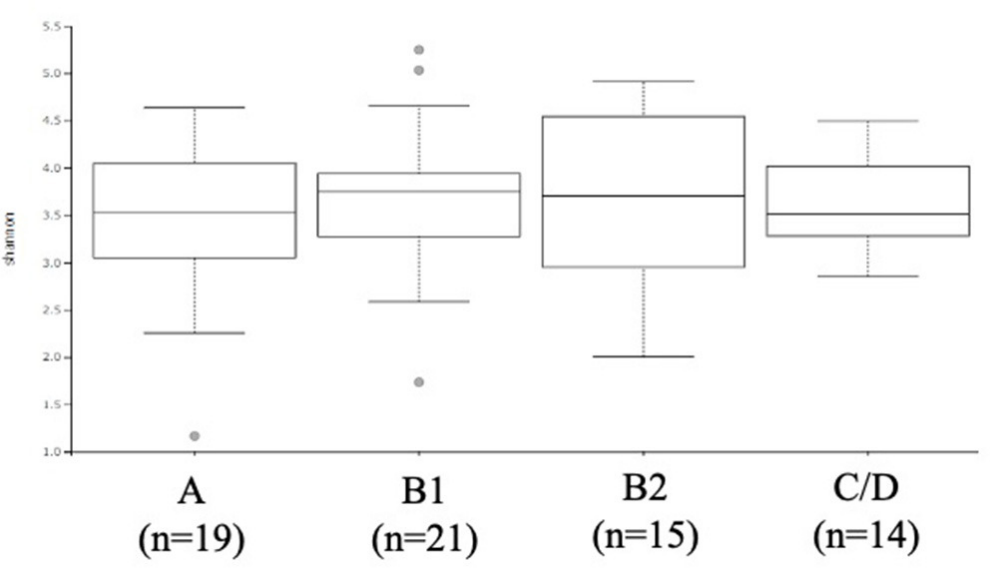
α-diversity: Shannon index based on operational taxonomic unit abundance of healthy Chihuahuas (H) and Chihuahuas with myxomatous mitral valve disease divided into three groups (B1, B2, and C/D).

**Figure 2:**
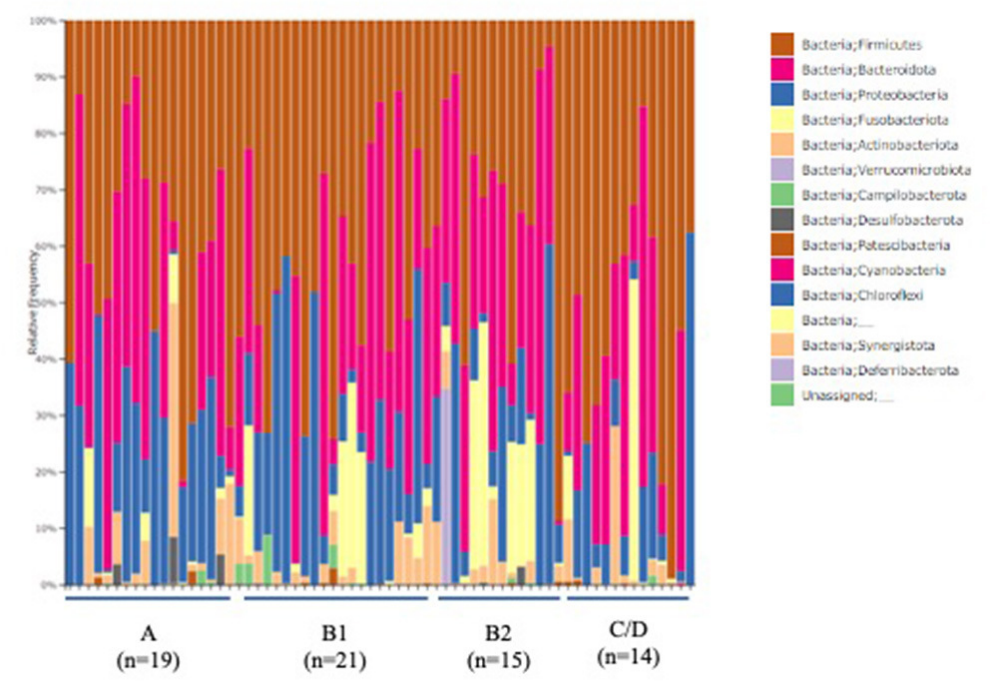
Abundances of taxa in the intestinal microbiota of healthy Chihuahuas (H) and Chihuahuas with myxomatous mitral valve disease divided into three groups (B1, B2, and C/D) (phylum level).

The ACVIM stage classification showed no significant difference in alpha diversity between the groups (Figure 1). In terms of the composition of the intestinal microbiota at the phylum level, most cases were dominated by bacterial species in the following order: Firmicutes, Bacteroides, Proteobacteria, Fusobacterium, Actinobacteria (Figure 2). There was no significant difference in the composition of the intestinal microbiota at the phylum level between the groups. Firmicutes/Bacteroidetes ratio was 82.6 ± 383.7.

## Discussion

As shown in Table 3, there was a tendency for the measurements obtained by echocardiography and chest radiography to worsen with increasing severity of MMVD. LA, LA/Ao, E vel, VHS, and VLAS were significantly increased in heart failure dogs compared with other groups, suggesting that they are important items indicating the severity of MMVD. LA/Ao and E vel have been reported to be important parameters that can be poor prognostic factors in MMVD, and the results of this study are consistent with previous reports (25). FS and LVEF did not differ significantly, although there was a trend toward increased values due to worsening MMVD. Worsening of MMVD has been reported in the past to increase FS, which was also true in this study, although the difference was not significant (26). FS is influenced by a variety of factors, including age, sex, breed, concomitant disease, preload, post load, and wall stress (20, 27–29). Also, many of the patients in this study were taking pimobendan internally. Pimobendan has been reported to increase FS and may have influenced this study (30). It has also been reported that the more severe the myocardial failure, the less FS that was elevated in MMVD (26). This study is difficult to evaluate because no histological examination of the myocardium was performed, but the influence of such a condition on the results should be considered.

**Table 3:** Echocardiographic and radiographic data in healthy Chihuahuas (H) and Chihuahuas with myxomatous mitral valve disease divided into three groups (B1, B2, and C/D).

	**H (*n* = 19)**	**B1 (*n* = 21)**	**B2 (*n* = 15)**	**C/D (*n* = 14)**	***P*-value for all**
LA (mm)	13.2 ± 2.2	15.2 ± 2.7	23.3 ± 6.1 ^b, d^	32.0 ± 7.4 ^c, e, f^	<0.001
Ao (mm)	9.41 ± 1.0	10.2 ± 1.2	10.9 ± 1.3 ^b^	10.5 ± 1.3 ^c^	0.005
LA/Ao	1.4 ± 0.2	1.5 ± 0.2	2.1 ± 0.5 ^b, d^	3.1 ± 0.9 ^c, e, f^	<0.001
IVSDd (mm)	5.2 ± 1.2	5.2 ± 0.8	4.6 ± 0.9	4.4 ± 1.2	0.062
LVPWd (mm)	5.4 ± 0.9	5.6 ± 1.0	5.1 ± 0.8	5.1 ± 1.2	0.292
LVIDd (mm)	17.0 ± 2.6	19.1 ± 3.4	25.5 ± 3.4 ^b, d^	27.5 ± 4.1 ^c, e^	<0.001
LVIDs (mm)	10.8 ± 2.6	12.0 ± 2.2	14.1 ± 2.3 ^b^	15.5 ± 3.6 ^c, e^	<0.001
LVIDDN	1.7 ± 0.2	1.8 ± 0.1	1.9 ± 0.1 ^b^	1.9 ± 0.2 ^c^	<0.001
E vel (m/s)	0.6 ± 0.1	0.7 ± 0.2	0.9 ± 0.2 ^b^	1.3 ± 0.5 ^c, e, f^	<0.001
A vel (m/s)	0.6 ± 0.2	0.8 ± 0.2 ^a^	1.0 ± 0.2 ^b^	0.8 ± 0.3	<0.001
MR vel (m/s)	nothing	5.7 ± 0.6	5.4 ± 0.5	5.3 ± 0.6	0.286
FS (%)	35.7 ± 6.3	36.0 ± 6.7	38.8 ± 12.9	44.4 ± 11.0 ^c^	0.037
LVEF (%)	68.3 ± 8.4	67.5 ± 7.6	72.0 ± 9.0	72.6 ± 11.7	0.338
VHS (v)	9.2 ± 0.9	9.4 ± 0.8 ^a^	10.6 ± 0.7 ^b, d^	11.5 ± 1.2 ^c, e, f^	<0.001
VLAS (v)	2.0 ± 0.2	2.3 ± 0.3 ^a^	3.1 ± 0.3 ^b, d^	3.8 ± 0.4 ^c, e, f^	<0.001

*Data are presented as mean ± standard deviation (SD).*

*LA, left atrium; Ao, aorta; LA/Ao, maximal diastolic left atrial body-to-aortic root diameter ratio in the right, parasternal, short-axis view of the heart base; IVSTd, interventricular septum thickness in diastole; LVPWd, left ventricular posterior wall thickness in diastole; LVIDd, left ventricular internal diameter in diastole; LVIDs, left ventricular internal diameter in systole; LVIDDN, left ventricular internal diameter in diastole, indexed to the bodyweight; E vel, early wave velocity; A vel, atrium wave velocity; MR vel; mitral regurgitation velocity; FS, shortening fraction; LVEF, left ventricular ejection fraction; VHS, vertebral heart score; VLAS, vertebral left atrial size.*

*P-value for all: Results of the overall ANOVA F-test.*

*Pairwise comparisons were made using the Tukey-Kramer test.*

*^a^Statistically significant difference between H and B1 (P < 0.05).*

*^b^Statistically significant difference between H and B2 (P < 0.05).*

*^c^Statistically significant difference between H and C/D (P < 0.05).*

*^d^Statistically significant difference between B1 and B2 (P < 0.05).*

*^e^Statistically significant difference between B1 and C/D (P < 0.05).*

*^f^Statistically significant difference between B2 and C/D (P < 0.05)*.

As shown in Figures 1, 2, there was no difference in α diversity or composition of the gut microbiota among the groups classified by the ACVIM guidelines in the gut microbiota analysis. In a study by Sandri et al. (31), the gut microbiota of dog was found to be composed of Firmicutes (43%), Bacteroidetes (19.8–26.9%), Fusobacteria (4.7–11%), and Proteobacteria (1.3–4.3%); in another study, it was found to be composed of Firmicutes (84% of all sequences), Bacteroidetes (2.9%), Fusobacteria (3.2%), Proteobacteria (7.8%) and Actinobacteria (1.7%) (32). These differences are thought to be due to individual and animal differences in intestinal microbiota (33). The intestinal microbiota of each case obtained by this study was consistent with previous reports in dogs. Human medicine has reported changes in gut microbiota composition in heart failure patients (6, 7). In this study, the FS and LVEF of heart failure dogs were normal and there was no obvious decrease in cardiac output. Decreased cardiac output has been reported to reduce blood flow to the intestinal tract and enteric membrane arteries, resulting in changes in the composition of the intestinal microbiota (6, 10). Considering the results of this study, it is possible that there is no obvious change in the composition of the gut microbiota in dogs with heart failure that do not have a reduced cardiac output. Next, we need to study the composition of the gut microbiota in dogs with heart failure due to MMVD with reduced cardiac output.

The intestinal microbiota is said to be affected by various factors such as individual differences and living environment, and it has been reported in dogs that it also changes with age. The age of Group H was lower in this study, and the difference in age between the groups may have affected the results. Chihuahuas are the preferred breed for MMVD, and the Chihuahuas that did not have MMVD tended to be sampled at a relatively young age (34–37). On the other hand, there is no significant age difference between the groups affected by MMVD (B1, B2, C/D), and comorbidities and medications administered need to be considered. The major difference was the presence of diuretics and the presence of CKD. In Group C/D, 9/14 patients were taking diuretics due to worsening MMVD, and in Group B2, 3/15 patients were taking diuretics. Diuretics in Group B2 were used to reduce cough and were used at lower volumes than in Groups C/D. In human medicine, chronic kidney disease has been reported to affect the intestinal microbiota (38). The accumulation of urea in the blood due to decreased renal function increases urea efflux in the intestine, and urease-containing intestinal bacteria increase ammonia concentration in the intestine, affecting the intestinal microbiota (39). The possibility of intestinal ischemia and acidosis due to uremia has also been suggested. We need to consider the possibility that the dogs in this study had various comorbidities (e.g., CAD, hypothyroidism, etc.) that may have affected the results.

Two cases were taking antibiotics and many cases were taking ACE-I internally. It has been reported that antibiotics and ACE-I affect the intestinal microbiota, which may have influenced the results of this study (40). In addition, the number of cases in group C/D was less than the other groups (H, B1, B2) in this study. It is necessary to consider the possibility that this may have affected the results.

The effect of diet on intestinal microbiota has been reported in dogs (33, 41). Specifically, dogs fed a natural diet have more diverse and abundant microbial composition in the gut microbiota than dogs fed a commercial feed. In this study, there was no uniformity in the diets, as shown in Table 2. In addition, the treatment diet contained several dietary fibers, including prebiotics. It is highly possible that the lack of uniformity in diet content and composition affected the gut microbiota, and the results of this study should be discussed in this light.

In this study, we were not able to confirm changes in the gut microbiota according to ACVIM stage. However, because this study was a clinical trial, there were a variety of factors in each case, and the influence of these factors on the results of this study cannot be measured. In the future, it is necessary to conduct a study with uniform conditions.

## Data Availability Statement

The original contributions presented in the study are included in the article/supplementary material, further inquiries can be directed to the corresponding author/s.

## Ethics Statement

The animal study was reviewed and approved by the Rakuno Gakuen University, School of Veterinary Medicine Institutional Animal Care and Use Committee (approval No. VH19A10). Written informed consent was obtained from the owners for the participation of their animals in this study.

## Author Contributions

RA: sample collection, data analysis, and writing the draft. KU and KI: sample collection and data analysis. AS, GI, MA, and TF: data analysis for gut microbiota. MI: study design and data analysis. All authors contributed to the article and approved the submitted version.

## Conflict of Interest

AS, GI, MA, and TF were employed by Anicom Inc. The remaining authors declare that the research was conducted in the absence of any commercial or financial relationships that could be construed as a potential conflict of interest.

## Publisher's Note

All claims expressed in this article are solely those of the authors and do not necessarily represent those of their affiliated organizations, or those of the publisher, the editors and the reviewers. Any product that may be evaluated in this article, or claim that may be made by its manufacturer, is not guaranteed or endorsed by the publisher.
